# Design and Validation of an FPGA-Based Configurable Transcranial Doppler Neurofeedback System for Chronic Pain Patients

**DOI:** 10.3390/s18072278

**Published:** 2018-07-14

**Authors:** Beatriz Rey, Alejandro Rodríguez, Enrique Lloréns-Bufort, José Tembl, Miguel Ángel Muñoz, Pedro Montoya, Vicente Herrero-Bosch, Jose M. Monzo

**Affiliations:** 1 Departamento de Ingeniería Gráfica, Universitat Politècnica de València, Camino Vera s/n, 46022 Valencia, Spain; alrodor@upv.es; 2 Instituto de Instrumentación para Imagen Molecular (I3M), Centro Mixto CSIC—Universitat Politècnica de València—CIEMAT, Camino de Vera s/n, 46022 Valencia, Spain; enllobu@gmail.com (E.L.-B.); viherbos@eln.upv.es (V.H.-B.); jmonfer@upvnet.upv.es (J.M.M.); 3 Departamento de Neurología, Hospital Universitari i Politècnic La Fe, 46026 Valencia, Spain; tembl_jos@gva.es; 4 Departamento de Personalidad, Evaluación y Tratamiento Psicológico, Universidad de Granada, 18071 Granada, Spain; mamuoz@ugr.es; 5 Instituto Universitario de Investigación en Ciencias de la Salud, Universitat Illes Balears, 07122 Palma, Spain; pedro.montoya@uib.es

**Keywords:** transcranial doppler ultrasound, neurofeedback, digital signal processing, chronic pain, fibromyalgia, FPGA, System on Chip

## Abstract

Neurofeedback is a self-regulation technique that can be applied to learn to voluntarily control cerebral activity in specific brain regions. In this work, a Transcranial Doppler-based configurable neurofeedback system is proposed and described. The hardware configuration is based on the Red Pitaya board, which gives great flexibility and processing power to the system. The parameter to be trained can be selected between several temporal, spectral, or complexity features from the cerebral blood flow velocity signal in different vessels. As previous studies have found alterations in these parameters in chronic pain patients, the system could be applied to help them to voluntarily control these parameters. Two protocols based on different temporal lengths of the training periods have been proposed and tested with six healthy subjects that were randomly assigned to one of the protocols at the beginning of the procedure. For the purposes of the testing, the trained parameter was the mean cerebral blood flow velocity in the aggregated data from the two anterior cerebral arteries. Results show that, using the proposed neurofeedback system, the two groups of healthy volunteers can learn to self-regulate a parameter from their brain activity in a reduced number of training sessions.

## 1. Introduction

Neurofeedback is a self-regulation technique that is applied to obtain voluntary control over activity in specific brain regions. The training is performed providing the subject with feedback about the activity in the brain region of interest. Research has shown that this approach has been effective for improving symptoms of patients with different types of disorders or alterations (such as depression, anxiety, post-traumatic stress disorder, affective disorders, epilepsy, brain injury, or degenerative motor disorders) [1,2]. If a specific disorder is associated with altered patterns of brain activity in specific brain regions, a neurofeedback training system can be designed with the goal of modifying the neural activity in those regions.

Neuroscience studies have found that there are specific brain regions that are activated during painful stimulation, such as the primary and secondary somatosensory cortices, the insula, the anterior cingulate and the thalamus, as well as prefrontal and parietal regions [3]. These regions are part of the pain neuromatrix, which presents a higher activation and a more altered dynamic in the case of chronic pain patients [4]. It has been found that neurofeedback training with EEG [5] or real time fMRI [6,7] can be useful for chronic pain patients

Another technique that has been applied to monitor brain activity responses associated with pain has been Transcranial Doppler Ultrasound (TCD). It is a diagnosis technique that registers non-invasively cerebral blood flow velocity (CBFV) in main cerebral arteries [8] and that has been widely applied in the psychophysiological field [9,10]. As the main arteries diameters remain without significant changes in a wide range of experimental conditions, the velocity variations that are registered using TCD reflect changes in regional cerebral blood flow generated by neuronal activity [11].

The technique is suitable for analyzing hemodynamical changes in the brain with a high temporal resolution complementing other brain activation monitoring techniques such as fMRI. Its spatial resolution is restricted to the brain regions supplied by the vessels that are being monitored. However, it is appropriate to distinguish between activations in cerebral regions irrigated by different arteries in each hemisphere.

In the case of pain studies, the main arteries that are monitored are the anterior cerebral arteries (ACA) and the middle cerebral arteries (MCA) [12,13,14]. The ACAs supply blood to the medial and superior areas of frontal and parietal lobes, which include regions associated with the processing of affective and cognitive components of pain, while the MCAs irrigate lateral regions of cerebral hemispheres, including the somatosensory cortex, which are more related with sensory aspects of pain [14,15]. Painful stimulation (both with temperature and pressure) has resulted in measurable variations in CBFV of the ACAs and MCAs [12,13,14]. In the case of chronic pain patients (fibromyalgia patients), these changes are even more pronounced than in control volunteers [13,14]. Changes in resting-state characteristics of CBFV have also been observed in chronic pain patients in comparison with the general population. The envelope CBFV from patients has a higher complexity and changes in the distribution of the power spectral density [16].

Taking these previous results into account, it seems logical to propose a configurable TCD-based neurofeedback system to train the altered parameters in chronic pain patients.

### 1.1. Related Work

To our knowledge, there is only one previous study that has checked that healthy volunteers can self-regulate TCD parameters [17]. In this previous work, the system was not configurable, as long as it was specifically focused on the mean CBFV in the MCAs and it had no possibilities for expansion, configuration, or re-design. In order to have access in real time to the envelope CBFV signals registered by the commercial TCD system, it was necessary to digitalize the analog output signals from the system using an analog to digital converter.

In the case of other technologies for neurofeedback, such as EEG, many systems work without any additional hardware to have access and process the registered signals. If real time access to the monitored signals is provided by the system, the processing can be conducted with existing applications such as BCI2000 [18] or self-made programs in Matlab or C. However, some studies have applied specific hardware configurations that include analog to digital converters and microcontrollers for EEG-based neurofeedback systems [19].

Focusing again on TCD-based systems, it is interesting to mention the advances that have occurred in recent years in the Brain computer interfaces (BCIs) research area. BCI research requires technological systems that share many design issues with neurofeedback ones. BCIs also need to have access in real time to the monitored signal and to process it in real time, although in the case of BCIs the calculated parameters are used to generate control commands that can be applied for different purposes such as controlling external devices [20]. In recent years, there have been many steps to advance into the design of a fully functional TCD-based BCI system. Most of these studies only include offline analyses that have been conducted to validate that it is possible to classify with sufficiently high accuracies between different mental states associated to the performance of very specific tasks [21,22,23,24]. As the analyses were conducted offline, TCD signals were recorded and later analyzed, so it was not necessary to have access to the signals in real time in order to process them. No specific hardware was needed to digitalize or analyze the recorded signals. The same happened with studies that proposed systems that combined TCD signals with fNIRS [25] or EEG [26] in order to increase classification accuracy. The features calculated by the previous BCI approaches were mainly based on temporal analyses, although some recent studies have also calculated statistical features based on a five-level wavelet decomposition [26,27].

The only studies that describe an online TCD based BCI system for controlling an onscreen keyboard [28,29] do not give details about how they have access to the TCD signal during the online procedure or how the signal is processed to obtain the input parameters for the classifier.

As a conclusion, it can be highlighted that the TCD-based neurofeedback or BCI systems that have been proposed and described in the previous studies are not based on hardware configurations that allow advances in the real-time acquisition and processing of the TCD signal. By default, these systems do not incorporate configurable hardware devices that can be applied to acquire and analyze the TCD signals from the participants in real time.

### 1.2. Research Goals and Contributions

Taking into account all the limitations of previous TCD-based neurofeedback and BCI systems, we propose a configurable system, based on FPGAs, with the possibility of applying advanced digital signal processing methods to the monitored signals in real time. Different parameters (from different domains: temporal, frequency, and information theory) and arteries can be monitored to provide the appropriate feedback to the user, and different training protocols can be applied from the application, allowing its personalized application for specific final users, such as chronic pain patients.

Most of the included parameters had not been considered in the previous TCD-based neurofeedback system [17]. Consequently, it has to be tested if it is possible to learn to self-regulate these parameters using the developed system. For ethical reasons, before analysing the clinical influence of the procedure on patients, the system has to be tested with healthy participants, to validate that it is working properly and to check that it is adequate for training the selected parameters. In all the procedure, we follow the recommendations from previous studies [30]. As they indicate, there is currently no single “correct” experimental design in neurofeedback studies, so “pilot testing” is necessary to adjust the system parameters. Furthermore, this testing should follow the methods of evidence-based medicine. The effects of neurofeedback should first be determined in healthy participants (i.e., [31,32,33]), before conducting any studies to analyze the clinical influence of the procedure on small patient groups.

Considering all these factors, the objectives of the present work are the following: (1) to technically design and develop a configurable TCD-based neurofeedback system; and (2) to validate the system with a small sample of healthy participants, analyzing if the trained participants are successful in the training of the selected parameters with different protocols.

## 2. Materials and Methods

### 2.1. Technical Aspects of the System

#### 2.1.1. Transcranial Doppler Monitoring

A commercial Transcranial Doppler ultrasound device is used to monitor CBFV (in cm/s) in ACA and MCA from both hemispheres: Multi-Dop T (DWL Compumedics Germany GmbH, Singen, Germany). In order to monitor CBFV from both hemispheres, two 2-MHz probes are used. Each probe can be fixed in the temporal bone of one hemisphere using a headset that is provided with the system. As each probe is designed to conduct the exploration simultaneously at two different depths, it is possible to monitor the ACAs (insonation depth between 65 and 70 mm) and MCAs (insonation depth between 50 and 55 mm) from both hemispheres at the same time.

QL software (DWL, Germany) was installed in the TCD ultrasound device and with the system it was possible to register the signals from the four cerebral arteries (ACA and MCA from both hemispheres). The registered signals are delivered at the analog output connector located at the rear panel of the device (dynamic range 2.5 V). The CBFV signals can also be locally saved in the TCD ultrasound device with a sampling rate of 100 Hz.

#### 2.1.2. Digitalization

A Red Pitaya board is used to acquire the four analog signals delivered at the analog output connector from the TCD ultrasound device. As indicated, the analog output connector generates a real-time analog copy of the CBFV acquired signals.

A self-made cable has been developed to interconnect the analog output from the TCD ultrasound device to the Red Pitaya ports.

Red Pitaya is an FPGA-based data acquisition board mainly used to develop your own measurement instrumentation equipment. An FPGA is a digital logic device that is composed of thousands of configurable interconnected logic cells. The FPGA can be configured to act as a digital complex system. FPGAs are mainly used for real time data processing and data analysis acceleration. The used FPGA is ISP (In System Programmable). Consequently, hardware can be reconfigured on the fly to increase its processing capabilities. Red Pitaya includes a Xilinx Zynq 7010 SoC FPGA together with two high speed ADCs (Analog to digital converters), two high speed DACs (Digital to Analog converters), 512 MB DRR3 SRAM memory block and different communication interfaces (USB, Gigabit Ethernet). Zynq 7000 FPGAs are System on Chip (SoC) devices that integrate together with the logical programmable logic a DualCore ARM Cortex-A9 microprocessor and 4 low speed analog-to-digital converters (ADC), with a 12-bit resolution and a sampling frequency of 100 kS/s. Its configurability, real time signal processing capabilities, the low cost of the system (around $300), and reduced dimensions (approximately 110 mm × 70 mm × 30 mm) are the main reasons to select this FPGA SoC board as the main processing block in the neurofeedback system.

The device simultaneously acquires data corresponding to the 4 CBFV channels using these low speed ADCs. Each ADC is configured to work with a sampling frequency of 48.45 kHz. As the acquired data are later decimated by 10, the resulting sampling frequency is 4845 Hz.

The hardware design has been made using the design tools from Xilinx, Vivado, version 2015.4 with webpack license. Several intellectual properties (IP) blocks were required for the design. The XADCWizard was used to configure the ADC: acquisition mode, channels and AXI-Stream bus interface to be used. Each sample is composed of 16 bits, but only 12 bits are effective. The remaining 4 bits are used to include the channel identifier for the current sample.

On the other hand, a DMA block is used to transform the information from non-mapped memory in the Stream bus, to fixed memory positions in the physical memory map of the FPGA (DDR3) memory that is shared by the Cortex-A9. The Cortex-A9 configures the logic hardware using software instructions that are sent through the AXI4-Lite bus, which interconnects the Cortex-A9 with the logic programmable blocks. Logic FPGA blocks act as memory-mapped peripherals at the microprocessor side.

The control software for the FPGA programmable subsystem that is executed in the Cortex-A9 has been written in C language. The operating system that runs in the Cortex-A9 is a Linux Debian.

**Figure 1:**
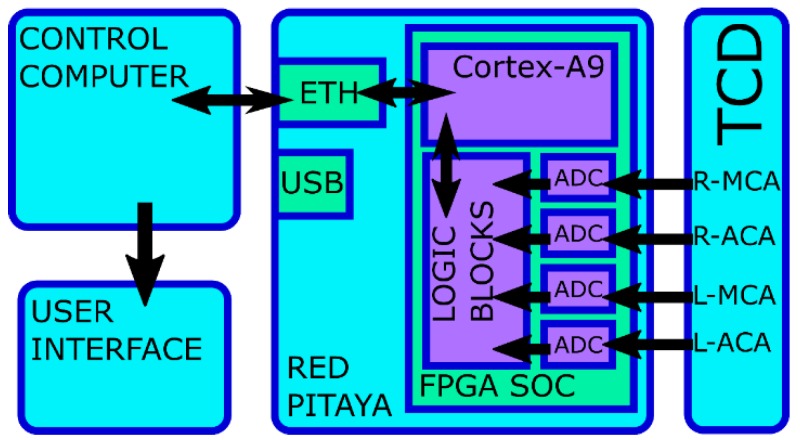
Block diagram with the different parts of the Transcranial Doppler Ultrasound (TCD)-based neurofeedback system.

The data frame size is fixed in 8192 bytes, that is, 1024 samples of 2 bytes per each of the four channels. When a complete data frame is generated, logic stores it in the shared memory. Then, they are read by the Cortex-A9 running software. The software constructs the UDP data frames and sends them to the computer (PC) using a socket. The PC is connected by Ethernet to the Red Pitaya board. The data link between the PC and the board can be USB, wireless or Ethernet, depending on the application requirements. Figure 1 presents a block diagram with the different parts of the TCD-based neurofeedback system.

#### 2.1.3. Data Processing

The system is prepared to obtain several parameters from the CBFV signal that can be used to provide feedback to the participant. The parameters are continuously calculated from the CBFV data contained in a buffer with a fixed and configurable length. This buffer is updated with new data as soon as it is received.

The first parameter that can be calculated is the mean CBFV.

The second parameter that can be obtained is the LZC. This feature evaluates the randomness of finite one-dimensional sequences [34], which is associated with the recurrence of the different substrings that appear in the signal [35]. Higher values indicate a higher complexity of the signal. This parameter has been included because it has been found that higher values of LZC from CBFV in L-MCA and L-ACA are observed in fibromyalgia patients in resting-state conditions in comparison with healthy subjects [16].

Finally, a parameter associated with the spectral distribution of the CBFV signal is obtained: the LF/HF ratio, which is defined as the ratio between the low frequency (LF: 0.04–0.15 Hz) and high frequency (HF: 0.15–0.4 Hz) components [36]. In this case, the parameter was included because previous research has obtained that, in resting-state conditions, this ratio (calculated from CBFV in L-MCA, R-MCA and L-ACA) is lower in fibromyalgia patients than in healthy controls [16].

#### 2.1.4. Neurofeedback Application

**Figure 2:**
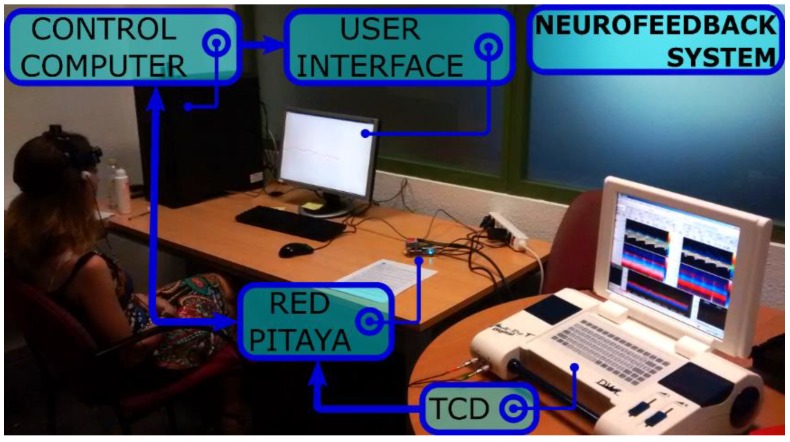
Different parts of the developed TCD-based neurofeedback system can be observed in the figure. The TCD ultrasound device registers the cerebral blood flow velocity (CBFV) signals from the participant. The analog output from this device is connected to the Red Pitaya board, which acquires the signals and delivers them to the control computer by Ethernet. The control computer receives the signals and executes the neurofeedback application. The feedback is provided to the participant through the computer screen (user interface).

In the computer, an UDP server is running to receive the data frames that are sent from the RedPitaya. An application was programmed using Matlab R2015b (The Mathworks Inc., Natick, MA, USA) to initialize the UDP server and to provide feedback to the participant about the relevant parameters of CBFV signals in the main cerebral arteries. The complete neurofeedback configuration that has been developed can be visualized in Figure 2.

As previously indicated, the neurofeedback application is configurable, and allows the experimenter to indicate the parameter that can be trained, selecting between LZC, ratio LF/HF and mean. This parameter will be calculated from the vessels that are chosen from the application: a single vessel (L-MCA, R-MCA, L-ACA and R-ACA) or a combination of vessels (both ACAs or both MCAs). In this last case, the CBFV from right and left hemisphere ACA or MCA are aggregated. The buffer length used to store the data before calculating the parameter can also be configured.

**Figure 3:**
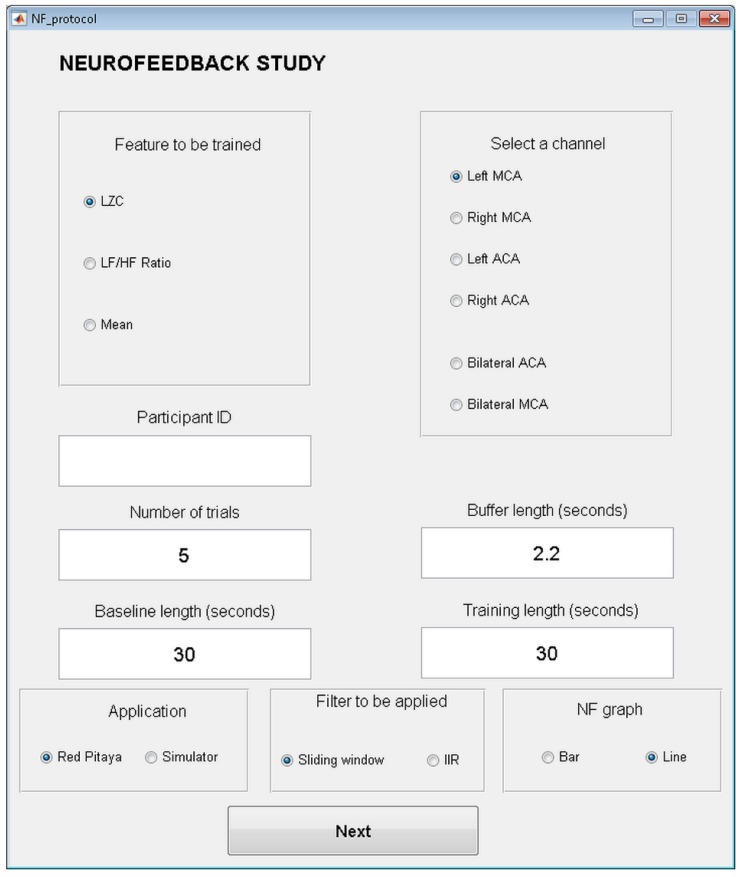
Configuration screen of the neurofeedback application.

On the other hand, the application also allows the experimenter to indicate how many trials are included in a training block. Each trial consists of a baseline period followed by training period, the length of which is also configurable. Finally, other options can also be selected: selecting to work in a simulation mode with files previously registered (instead of receiving in real time the acquired signals from the Red Pitaya), the filter to be applied to the signal (sliding window or IIR), and the kind of graph to be used to give the feedback to the user (Bar or line). All the different options and parameters that can be configured can be observed in Figure 3.

**Figure 4:**
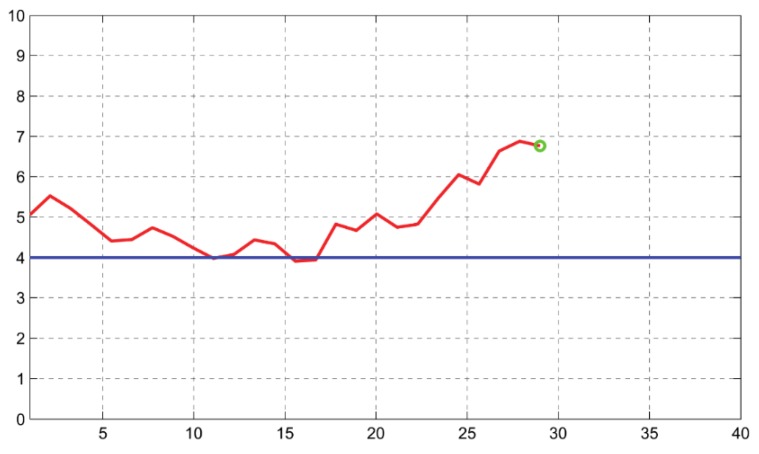
Graph shown to the volunteers during the neurofeedback training. An example from one of the training periods is shown in the figure. The goal that the participant is to achieve is that the red line goes below the 4 value, which is represented in the screen with a blue line. When the selected parameter is the mean CBFV, the 4 value represents a CBFV (cm/s) that is 3% smaller than the mean CBFV (cm/s) obtained during the baseline period. The current value of the parameter is marked in the graph with a green circle.

The selected parameter is represented on a figure on the computer screen to provide the visual feedback to the final user. This figure is a linear graph representing the parameter that is being trained. This parameter has to be calculated from the CBFV values (in cm/s) contained in the buffer window defined in the application, which is continuously being updated as new data is received. Initially, just a single point is represented, and every 1.1 s a new point is calculated and added on the right part of the graph. In order to smooth the representation and to avoid abrupt changes, the represented value is a weighted arithmetic mean of the current value and the three previous values. An example from one of the training periods of one participant is shown in Figure 4.

The vertical axis of the graph ranges between 0 and 10. A value of 5 corresponds to the mean value of the parameter during the baseline condition. Changes by 1 point (positive or negative) correspond to percentage changes (increases or decreases) in the trained parameter of 3% with respect to the parameter in the baseline period. This scale has been chosen following the arrangements made in the previous study with TCD-based neurofeedback [17].

**Figure 5:**
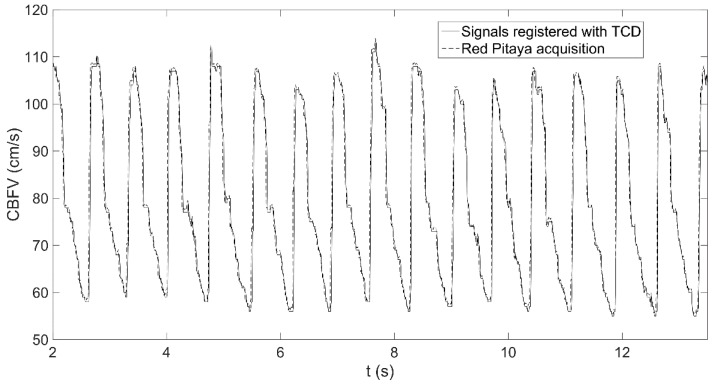
Representation of a fragment of the CBFV signal (cm/s) in the l left anterior cerebral artery (L-ACA) of a volunteer, as registered with the TCD ultrasound device (continuous line) and as acquired by the Red Pitaya (dashed line).

Before validating if the system could be successfully applied to train the selected parameters, it was checked that the acquisition system was working correctly. In order to do that, the Intraclass Correlation Coeficient (ICC) Cronbach α was calculated between a signal registered with the neurofeedback system and the original signal registered with the TCD system. A high reliability estimation between the two signals was obtained (α = 0.977). A representation of a fragment of both CBFV signals can be observed in Figure 5.

### 2.2. System Validation

A study was designed to test the developed system and evaluate if it could be successfully applied to train a parameter that has shown alteration in the CBFV responses of patients with chronic pain. The parameter that was selected was the mean of the aggregated CBFV in the ACAs.

This parameter was selected based on previous studies that have found an altered CBFV response in those arteries [13,14]. This altered response could be associated to medial structures that intervene in the processing of affective and cognitive aspects of pain.

Participants were randomly assigned to one of the two different versions of the training protocol that were proposed and that are described below: (1) long training periods: this protocol was composed of a reduced number of repetitions of long training periods (2 min); and (2) short training periods: this protocol included several repetitions of short training periods (30 s).

Prior to the proposal of the two protocols, some pilot testing was conducted to evaluate the effects on the training results of different period lengths, and longer training periods were avoided because of the difficulties of the participants for maintaining their training strategies during longer periods of time.

#### 2.2.1. Participants

As indicated in the Introduction, the validation of the system only requires a limited number of healthy participants [30]. In the current study, 6 volunteers have participated: 2 men and 4 women, aged between 18 and 66 years (mean age 39 years; SD 19.6). Three of the volunteers were assigned to one of the proposed training protocols in the present study, and the other three to the other training protocol (training protocols will be described in detail later). That gives us the possibility of evaluating the evolution of the success of different individuals during the training procedure with each protocol.

Exclusion criteria included major psychiatric diseases, neurological disorders, or relevant physical diseases. In order to recruit the volunteers, announcements were posted in notice boards of universities.

All subjects gave their informed consent for inclusion before they participated in the study. The study was conducted in accordance with the Declaration of Helsinki, and the protocol was approved by the Ethics Committee of the Universitat Politècnica de València (Project identification code: PSI2013-48260-C3-2-R).

#### 2.2.2. Questionnaires

Manual preference of the participants was assessed using the Edinburgh Handedness Inventory [37,38]. Besides, in order to evaluate their mood, the State-Trait Anxiety Inventory (STAI) questionnaire was applied [39]. Finally, after each session, participants had to answer a question describing the strategies that the participants had applied during the training.

#### 2.2.3. Experimental Design

Sessions were repeated several times in different weeks (4 different sessions in the first protocol and 3 different sessions in the second protocol).

Before starting the first session, participants received written information about the procedure and signed the informed written consent.

In the first session of the procedure, the volunteers filled out the complete STAI questionnaire and the Edinburgh Handedness Inventory. In the successive sessions, the volunteers just had to fill out the STAI-State questionnaire.

The Doppler box and the probes were adjusted to detect the ACAs by an experimented neurologist, and blood pressure was measured using a wrist blood pressure monitor (R3 Intellisense; Omrom Healthcare Co., Ltd., Kyoto, Japan).

After that, the training period started. The participants had been informed that the graph on the computer represented a parameter obtained from CBFV signals in a pair of cerebral vessels. Their task consisted of trying to reduce the parameter below the 4 value (see Figure 4) in the vertical axis (which corresponded to a decrease of 3% with respect to the CBFV mean during baseline). Following recommendations from previous works that remark that the use of explicit techniques is unnecessary [40,41], participants did not receive any indications about how to use specific strategies to control the CBFV parameter, so participants were free to try the mental strategy or cognitive scheme that is more suitable for them.

In the first protocol (long training periods), there were 6 trials in each session. Each trial was composed of a baseline period (2 min) followed by the training period itself (2 min). As there is only one previous study with TCD-based neurofeedback [17], this protocol is based on the protocol that was used in this previous study, where long training periods of several minutes were applied.

However, other hemodynamical techniques, such as real-time fMRI, have also been applied in neurofeedback studies [2,42]. Most real-time fMRI studies use a block design for the training. That means that they have to regulate the BOLD signal for periods of approximately 15–30 s, which alternate with resting periods with a similar duration. Usually, each trial includes a number of blocks between 3 and 6, and is repeated between 2 and 5 times in an experimental session [43].

These protocols based on shorter periods of approximately 15–30 s are also suitable for TCD-based neurofeedback, as TCD is also a modality to monitor hemodynamical changes in the brain (in this case, CBFV). That led us to include a second protocol (short training periods) in the experimental design.

In the second protocol (short training periods), there were 4 trials in each session. Each trial included 5 repetitions of the complete training period, which is composed of a baseline period (30 s) followed by a neurofeedback period (30 s). If the objective of reducing the parameter below the value of 4 was achieved, a congratulation message was shown on the screen during a short period of 5 s to make them know that they have succeeded.

**Figure 6:**
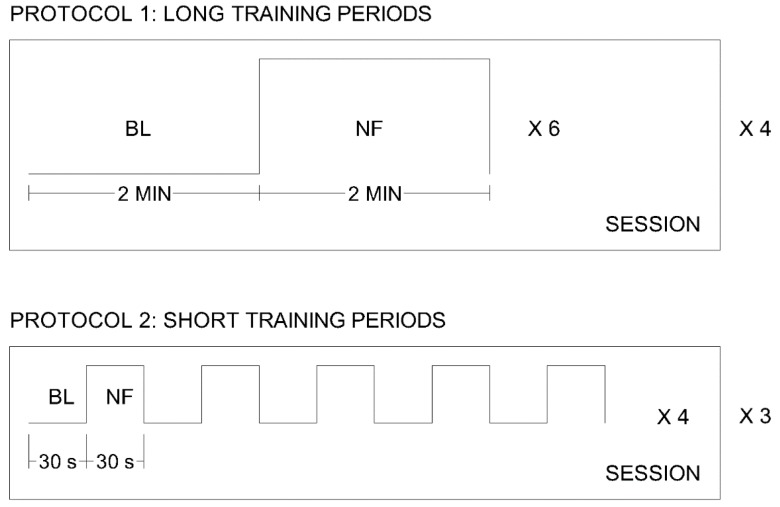
Schema of the two protocols applied in the validation sessions. Protocol 1 is composed of 4 sessions, with 6 trials in each session. A trial is composed of 2 min of baseline (BL) and 2 min of neurofeedback training (NF). Protocol 2 is composed of 3 sessions, with 4 trials in each session. A trial is composed of 5 repetitions of a 30-s baseline period (BL) and a 30-s neurofeedback period (NF).

A graphical schema describing the two protocols can be observed in Figure 6.

During any baseline period, participants were told to relax and keep their eyes open looking to a white cross shown in the center of the screen.

After having finished the training period of each trial, blood pressure was measured again. Finally, the participant had to answer the question about the strategies that he or she had applied during the training.

#### 2.2.4. Success Level

The success level, defined as the percentage of successful training periods in each session, was calculated. A training period was considered successful if the mean CBFV has descended a 3% (the graph has descended below the 4 value) during the training period with respect to the mean CBFV during the previous baseline.

## 3. Results

### 3.1. Questionnaire Data

All the participants were right-handed based on the Edinburgh Handedness Inventory responses. None of them presented high levels of trait anxiety. Furthermore, the participants did not show high levels of state anxiety in any of the sessions in which they participated. Details for each group of participants (depending on the protocol that they followed) can be observed in Table 1.

From the responses to the question about the strategies that participants had tried during the session, it could be observed that they had applied different techniques to train the parameter, such as thinking of happy songs, thinking of annoying things, focusing on the changes of point position in the graph representation, focusing on breathing patterns, arithmetic tasks, or avoiding thinking about the task performance.

### 3.2. Success Level

**Figure 7:**
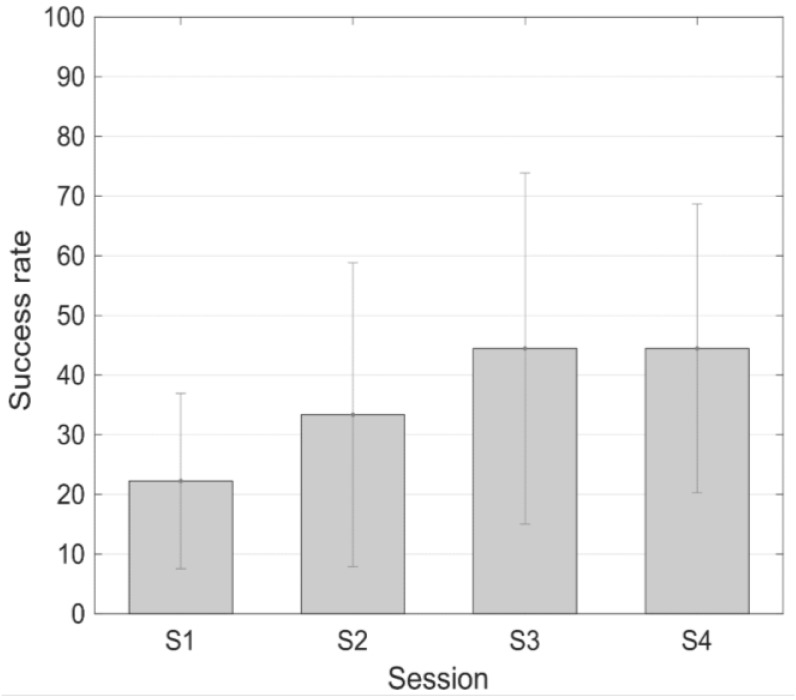
Success rate (mean ± standard error of the mean) during the different sessions of the protocol with longer training periods.

The success level from the three subjects that followed the protocol with long training periods is represented in Figure 7. In the first subject there is an increment in the success rate from 16.66% in the first session to 83.33% in the last session. The second subject did not achieve a successful training. And the third subject’s success rate increased from 0% in the first session to 50% in the last one.

**Figure 8:**
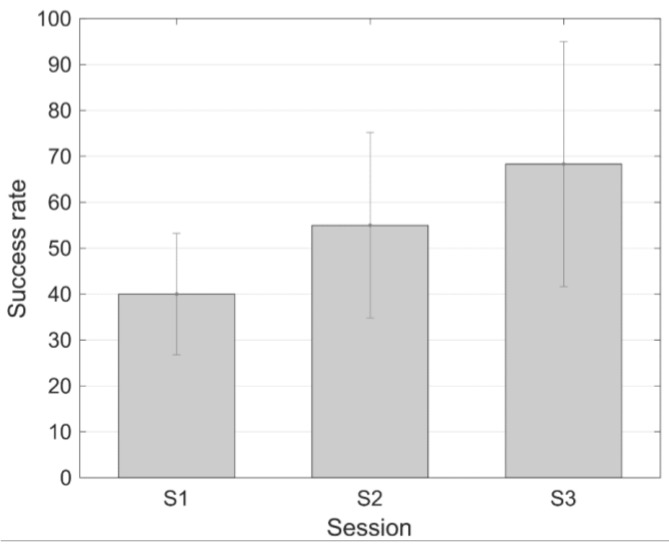
Success rate (mean ± standard error of the mean) during the different sessions of the protocol with shorter training periods.

Analogously, Figure 8 shows the task performance success level corresponding to the three subjects that followed the protocol with short training periods. In this case, the first subject did not achieve a successful training. But the other two subjects improved their success rate from 15% (subject 2) and 45% (subject 3) in the first session, and to 95% in both cases in the last session.

Blood pressure values from each participant remained stable during the procedure.

## 4. Discussion

In this work, a new and configurable TCD-based neurofeedback system has been designed and developed. The system allows the selection of aspects such as the parameters that are trained by the system, the vessels from which they are calculated and the length of the different periods of the training protocol. That way, it is possible to personalize the neurofeedback protocol, making it more suitable for specific groups of users, such as patients with chronic pain.

One of the hardware parts that gives the system the needed flexibility and power is the acquisition system based on the Red Pitaya board, which is a device with a reduced size and cost which includes a Xilinx FPGA (Xilinx Zynq 7010 SoC) with a DualCore ARM Cortex-A9. This hardware combination provides several advantages to the board. It improves in different ways traditional neurofeedback microprocessor-based data acquisition systems [44]. The approach presented in the present work increases the real-time data processing capabilities of the system, allowing us to process the acquired signals in a faster way to extract important parameters. This can be done because FPGA configurable hardware is used to generate dedicated logic to implement the complex processing algorithms. Besides, having a board with an FPGA and a DualCore ARM Cortex-A9 gives processing power to the system. It is possible to apply parts of the digital signal processing from the FPGA and parts from the Cortex-A9. Although both systems can perform digital signal processing operations, the CPU is more appropriate to run control software and the FPGA to execute complex signal processing in real-time.

The system, as a configurable logic hardware, allows us to interact or to synchronize the acquired signals with signals from other laboratory subsystems in real-time. The board has different input-output connectors that make the device accessible from different kinds of systems. In our case, a specific cable has been developed to connect the input connectors to the analog outputs from the Doppler device. Having 4 ADCs allow the simultaneous acquisition of up to 4 CBFV channels from the DWL Doppler Box, making it possible to simultaneously acquire data from both ACAs and both MCAs if necessary. But, in the future, other configurations could be easily prepared in case there is a change in the Doppler device that is applied or if signals from other devices have to be monitored.

The nature of the ISP system allows the modification of the hardware configuration at any time introducing more functionality or new processing algorithms. For this reason, it is an ideal research platform to test new real time processing algorithms with the goal of improving the neurofeedback functionality. New developed research functionalities and algorithms can be reconfigured easily on the daily used neurofeedback systems installed on hospitals or on research laboratories. This reconfiguration can be performed in remote mode because the developed neurofeedback system can be connected to the institution network where it is installed.

Moreover, digital hardware FPGA design was a complex task traditionally reserved to electronic engineers. However, nowadays, new FPGA logic synthesizers allow us to generate hardware logic from a behavioral algorithm description in C++. These new logic synthesizers permit that non electronic engineer researchers can easily design FPGA logic to improve the processing capabilities of their neurofeedback systems.

Another thing to take into consideration is the portability of the system due to the small size of the acquisition system and its different connectivity possibilities. In the future, the control PC can be replaced with a tablet or smartphone connected directly to the Red Pitaya board, thus reducing the neurofeedback system to the TDC, the Red Pitaya board and the two tablets or smartphones (one to control the system and another one as a user interface)

All these aspects show that the hardware and software selection that has been made for the design and development of the neurofeedback system is adequate and constitutes an advance with respect to the only previous TCD-based neurofeedback system [17], which was not configurable and only allowed the training of one specific parameter which was the mean CBFV in MCAs, or with respect to previous TCD-based BCI systems [21,22,23,24,25,26,27,28,29].

The proposed TCD-based neurofeedback system can be adapted to be applied with different kinds of final users, but it has been specifically designed for chronic pain patients to reduce their pain perception. The advances and the scientific evidence gathered during the last years regarding the neural bases of chronic pain allow us to include in the system the possibility of training several parameters in which alterations have been found in chronic pain patients [13,14,16]. The specific parameters that have been selected are the mean CBFV, the LZC, and the ratio LF/HF of the ACAs.

It is important to remark that TCD-based systems provide a small spatial resolution, which is determined by the brain areas supplied by the vessel under study. This impossibility of training CBFV in smaller brain areas is a limitation of any TCD-based neurofeedback system.

Focusing on the designed system, it is necessary to evaluate if it is possible to learn to self-regulate one of these parameters. So this possibility has been tested with a small group of healthy volunteers with low levels of anxiety as evaluated with the STAI questionnaire. Results have shown that the volunteers can be trained to self-regulate a CBFV parameter in a reduced number of training sessions.

Furthermore, there are some relevant observations that have been made and that may be necessary to adapt and apply the training protocol for chronic pain patients.

On one hand, it was not considered necessary to give explicit instructions to the participants about the mental strategies to be applied to achieve a successful training. The results from the validation show that several strategies have been applied by the different participants. And successful training results have been observed with highly different training strategies. This is coherent with previous works that remark that explicit techniques are unnecessary [40,41].

One of the problems of neurofeedback is that an effective mental strategy may show comparable effects regardless of how it has been learned: through neurofeedback training or just because the experimental procedure (consisting of a repetitive training with simple instructions) may lead the participant to be in a specific mental state. However, as in the present study participants had to answer to a question describing the strategies that they had applied during the training, it could be checked that different strategies were applied depending on the participants and that none of them mentioned strategies that could be generated indirectly by the experimental procedure such as relaxation.

On the other hand, it is important to highlight that two training protocols have been assessed in the present work. One training protocol is pretty similar to the protocol applied in the previous work with TCD-based neurofeedback [17], with long training periods of around 2 min, and the other one is based on shorter training periods (30 s) with several repetitions. This second protocol is more closely related to those applied if neurofeedback based on other techniques such as real-time fMRI is used [42]. The mean success rate increases with the number of sessions with both protocols, with higher improvements (higher success rates and faster learning curves) using the protocol with shorter training periods. The mean success rate in the last session of the training with longer training periods is 44.44%. In the case of the protocol with shorter training periods, the mean success rate in the last session is 68.3%. It can be observed that a higher increment is observed in the protocol with shorter training periods.

In a similar way to studies with brain–computer interfaces [45], the success rate parameter has been defined to evaluate if the participant can self-regulate the trained parameter during the different parts of the training protocol. In the current system, a training period is considered successful if there has been a 3% decrement in mean CBFV with respect to the mean CBFV value in the preceding baseline. This percentage change value has been selected based on observed variations in the different training sessions from the previous TCD-based neurofeedback work [17].

In the second protocol (short training periods), if the participant was able to reduce the parameter below the 4 value during a neurofeedback period (30 s), a congratulation message was shown on the screen for a short period of 5 s to make him/her know that he/she has succeeded. There are neurofeedback researchers that indicate that the amount of positive feedback participants receive has an influence on the training outcome [46]. This is probably one of the reasons that justify the faster learning curve and higher success rates that are observed with the second protocol.

Furthermore, it has been observed during the trials that participants show greater difficulties in maintaining the training during longer periods. Reducing the training times from 2 min to 30 s, makes it easier for the participants to maintain the mental strategy they are applying during the whole training period (30 s). Besides, it also allows more repetitions of the training period, which probably also has an influence on the final learning curves.

Besides, results have shown that there is a high heterogeneity in controls and that not all of them can learn to self-regulate correctly the trained parameter. That is the case for subject 2 of the protocol with longer training periods and for subject 1 of the protocol with shorter training periods. This is coherent with previous scientific studies, which have found that, even with different training protocols and different experimental conditions, there exists approximately 20% of individuals that cannot self-regulate their brain activation [45]. Cerebral plasticity is an important factor that may explain observed differences between participants. There may be factors related to the specific cerebral plasticity of each participant that can have an influence on the effectivity of the training.

This heterogeneity is expected to appear in chronic pain patients, or even to be greater, as long as these patients show altered brain activations in their pain neuromatrix, so it will be necessary to examine the number of training sessions required for chronic pain patients in order to learn to self-regulate a specific parameter. On the other hand, the influence of this self-regulation on clinical parameters such as the chronic pain level and the intensity of the patient symptoms has to be evaluated in future studies, as the main goal of this kind of system is to achieve clinical and significant improvements on patients’ pain perception.

There may be factors related to the specific cerebral plasticity of each patient that can have an influence on the effectivity of the training. Deepening on the study of these factors may allow the use of neurofeedback in the way of personalized medicine, defining the characteristics of the intervention and its possibilities. Methodological aspects [43] such as the number of sessions, interval between sessions, or the trained parameters should be adjusted based on the personal characteristics of each patient.

Including FPGA systems is a way to advance in a research area. These configurable devices allow us to apply complex digital signal processing methods to the monitored signals in real time, which cannot be applied using traditional hardware configurations in real time.

The originality of the present work is that it is designed as a configurable system, based on FPGAs, with the possibility of applying advanced digital signal processing methods to the monitored signals in real time, thus overcoming the limitations from previous studies that do not incorporate this kind of device. Different parameters (not only from the temporal domain, but from other different domains: temporal, frequency and information theory) and arteries can be monitored in the proposed configurable TCD-based neurofeedback system.

Taking all the previous comments into account, it can be concluded that the application of a neurofeedback system for chronic pain patients is highly relevant for researchers interested on cerebral plasticity, neuroscience, or neurorrehabilitation. The configurable neurofeedback system presented in this work provides all the necessary hardware, software, and all the configuration capabilities required to train specific subjects to self-regulate their brain activity parameters.

## Figures and Tables

**Table 1:** State-Trait Anxiety Inventory (STAI)-Trait, STAI-State during the first session of the training (PRE) and STAI-State during the last session of the training (POST). Data are presented as mean value ± standard deviation.

Protocol	STAI-Trait	STAI-State PRE	STAI-State POST
Long training periods	8.33 ± 14	13.67 ± 11.23	11 ± 2
Short training periods	14 ± 1.73	17.33 ± 8.14	17 ± 8.88
